# Rational design of k-casein peptides to modulate GSK-3B dynamics for Alzheimer’s therapy

**DOI:** 10.1038/s41598-026-42103-z

**Published:** 2026-03-09

**Authors:** Neda Moghaddam, Ali Ramazani, Armin Zarei

**Affiliations:** 1 The Organic Chemistry Research Laboratory (OCRL), Department of Chemistry, Faculty of Science, University of Zanjan, Zanjan, 45371-38791 Iran; 2 The Convergent Sciences & Technologies Laboratory (CSTL), Research Institute of Modern Biological Techniques (RIMBT), University of Zanjan, Zanjan, 45371-38791 Iran; 3 Universal Scientific Education and Research Network (USERN), University of Zanjan, Zanjan, 45371-38791 Iran

**Keywords:** K-casein, Peptide, GSK-3β, Alzheimer’s, Neuroprotection, Biochemistry, Computational biology and bioinformatics, Drug discovery

## Abstract

GSK-3β is an important therapeutic target in Alzheimer’s disease due to its central role in tau hyperphosphorylation, and synaptic dysfunction. In this study, a κ-casein-derived peptide (LALTLPFLGA) was identified via HADDOCK and introduced to MD simulations for MM/PBSA per-residue analysis, revealing four unfavorable mutation sites (L12, L14, T15, F18). Using MCSM and ΔΔG-based substitution, a 48-peptide library was generated; 22 candidates satisfied toxicity/allergenicity filters. Docking and MD simulations prioritized four leads—PEP8, PEP36, PEP40, and PEP44—with PEP8 (− 89.1 kcal·mol⁻¹) and PEP44 (− 88.4 kcal·mol⁻¹) as top binders. These mutants, all carrying L12H/L14H substitutions, demonstrated stronger HADDOCK scores (− 89.1 to − 84.6 kcal/mol) compared with the template. Additionally, PEP8 carried F18→Asp, and T15→Leu/Ile developed affinity in other mutants. PEP8 established 21 contacts, followed by PEP44 (19), PEP40 (17), and PEP36 (15), indicating that larger interaction networks are vital for binding. PEP8 contacted with the ATP-binding pocket (Val135, Thr138, and Tyr134), whereas PEP44 contacted catalytic Asp200. MD results also showed reduced RMSD, slightly lower Rg/SASA, and tighter PCA clustering, exhibiting that PEP8 and PEP44 induced compaction and restricted conformational freedom, and possibly, hindering substrate binding. MMPBSA confirmed PEP44 and PEP8 as strongest complexes (− 188.482 and − 184.404 kJ/mol), governed by van der Waals and electrostatic contacts, while PEP36 suffered solvation penalties and PEP40 remained hydrophobic-driven. Together, our computational pipeline highlights PEP8 and PEP44 as promising κ-casein-derived inhibitors of GSK-3β, illustrating a rational design of food-derived peptides as potential multifunctional candidates for Alzheimer’s disease.

**Supplementary Information:**

The online version contains supplementary material available at 10.1038/s41598-026-42103-z.

## Introduction

GSK-3β has appeared as an important pharmacological receptor for the development of novel therapeutics for Alzheimer’s disease (AD) because of its pivotal involvement in disease progression^1–3^. This protein is believed to promote excessive tau phosphorylation, a process that directly contributes to the formation of neurofibrillary tangles (NFTs), a defining neuropathological feature of AD. These insoluble aggregates significantly damage neuronal communication and plasticity, finally leading to cellular degeneration and cognitive deterioration^1,2,4^. Escalated activity of GSK-3β has been tightly related to tau dysregulation, structural disintegration of neurons, and the acceleration of neurodegenerative mechanisms^5^. In addition to driving tau pathology, this enzyme has also shown a crucial impact on amyloidogenic pathways in AD. Actually, GSK-3β regulates the proteolytic processing of APP (amyloid precursor protein), and facilitates the formation of amyloid-beta, causing the Aβ plaques generation in AD brains^6^. Furthermore, excessive function of the kinase also lies in the disruption of synaptic integrity and attenuation of long-term potentiation^7^, both essential mechanisms underlying memory formation and cognitive resilience. These phenomena demonstrates the potent role of GSK-3β in modulating synaptic efficacy, and thus, suppression of this enzyme activity may decline the mentioned neurocognitive deficits. More importantly, the insulin signaling within the central nervous system is believed to be negatively regulated by GSK-3β. Therefore, disrupted insulin pathways and resistance are established Alzheimer’s risk factors, so regulating GSK-3β activity might help reinstate signaling fidelity and alleviate neurocognitive deterioration linked to disease progression^8–10^.

Peptide pharmaceuticals have aroused many enthusiasts over the last couple of years as promising interventions across a spectrum of disorders such as oncology^11^ and metabolic disorders^12,13^. Their unique pharmacological profile—such as structural versatility, readily modifiable structure, and efficient cellular and tissue permeability—provides clear merits over conventional monoclonal antibodies and small molecules. Notably, their inherent biocompatibility and minimal immunogenicity enhance their suitability for the treatment of neurodegenerative disorders, particularly in AD. Peptides for Alzheimer’s therapy function via multiple pathways: preventing amyloid-beta buildup, suppressing brain inflammation, reducing oxidative damage, and decreasing tau-related abnormalities—offering promising multi-target strategies against Alzheimer’s pathology^14,15^.

Previous studies have examined the role of milk-derived substances in both detecting and managing AD, revealing that a regular consumption of dairy products might lower dementia risk^16–19^. Recently, it was demonstrated that goat milk induces protective effects on the nervous system and helps improve memory impairments in animals and humans. The positive outcomes might be associate with enhanced glutathione production along with reductions in total serum cholesterol, acetylcholinesterase activity, and malondialdehyde^20^. Moreover, specific peptides, βb-casomorphin-7 and βb-casomorphin-5, obtained from milk have exhibited promising activities against AD by suppressing acetylcholinesterase^21^. Recent study on AD mouse models also revealed that a protein found in milk, casein, may offer convenient benefits for mitigating memory loss^22^. A category of phosphoproteins found in casein demonstrates unique roles across food chemistry and nutritional biochemistry. The principal isoforms— αS2-, κ-, αS1-, γ-, and β-caseins—indicate various physicochemical peculiarities and biological roles. Representing nearly 10–12% of cow milk casein, κ-casein plays a crucial role in micelle stabilization by creating a barrier that inhibits aggregation behavior^23^. Although numerous biomedical tactics for AD have been proposed, peptidic fragments based on κ‑casein remain largely unexplored as GSK-3β inhibitors, highlighting a novel therapeutic research direction.

Computer-based techniques, such as MD simulations and molecular docking, are extensively used in the mysterious world of AD research, providing fast and cost-effective insights into therapeutic mechanisms^24,25^. These simulations offer beneficial information to expand our knowledge on interaction strengths, expose conformational dynamics, and identify critical amino acids influencing enzymatic activity, thereby supporting targeted pharmaceutical development to address the intricate, multifaceted pathology underlying Alzheimer’s disease progression. In this context, computational approaches have been extensively applied to explore food-derived peptides as novel GSK3-β inhibitors, providing potent therapeutic potential for AD through the rational design of naturally sourced inhibitory candidates.

## Materials and methods

### Peptide library design

In order to generate the peptide library, the κ-casein (Uniprot: P02668) as the template sequence was initially docked into the GSK-3β ATP-binding site using HADDOCK webserver for finding a 10-lneght peptide template. To reach this, the fasta sequence of the κ-casein (Uniprot: P02668) was introduced to AlphaFold2 to build a reliable 3D structure of the κ-casein. Afterward, the modelled κ-casein and GSK-3βs’ structures (PDB ID: 1Q5K) were subjected to the HADDOCK webserver, and the κ-casein– GSK-3β complex conformation corresponding to the docking cluster with the minimal binding energy was selected for further analysis. The target peptide template (L12A13L14T15L16P17F18L19G20A21) was then subjected to MD simulations to find the spots (if any) in the template sequence that are prone to mutations using the calculation of the MM/PBSA of each residue (Table 1). After finding the mutation spots, and the supplementations of the mutations to the peptide sequence, the peptide library was generated (Table**S1**,Supporting Information). Finally, all of the 3D structures of the peptides were generated using CHIMERA 1.8.1.

### Peptide–target docking studies

To evaluate peptide-mediated inhibition of GSK-3β in comparison to ATP, molecular docking studies were fulfilled using the HADDOCK platform. Peptide structures and GSK-3β (PDB: 3Q3B, chain A) were submitted, and the peptide–enzyme complex conformations with minimal docking scores were selected for interpretation.

### Toxicological and allergenic assessment

The assessment of toxicity and allergenicity of peptides is a vital step in developing safe anti-Alzheimer’s candidates, particularly since this neurodegenerative condition predominantly impacts older individuals who are often more vulnerable to harmful immune reactions. Severe responses might compromise therapeutic effectiveness and escalate the likelihood of side effects. Thus, toxicity and allergenicity assessments were conducted via ToxinPred and AllerTOP platforms since these online servers utilize machine-learning approaches to identify problematic peptide sequences during early-stage screening.

### Molecular dynamics (MD) simulations

MD simulations were employed to assess the behavior of peptide–GSK-3β complexes, following protocols consistent with prior methodology, including parameter sets, solvation details, and equilibration procedures. Simulations were executed via GROMACS version 2024.2, utilizing the CHARMM27 force field to prepare topological and coordinate information for both peptides and protein. Each cubic simulation box was solvated with the SPC/E water, ensuring a 1 nm buffer from the boundaries under periodic boundary conditions. System neutrality was achieved by introducing appropriate quantities of counter ions. Following this, energy minimization was supplemented by the steepest descent algorithm until energy is reached below 10 kJ/mol^26^. Subsequent equilibration occurred at 300 K using the Berendsen thermostat and maintained 1 bar pressure under both NVT and NPT ensembles. A 200 ns production trajectory was then computed employing the Particle Mesh Ewald (PME) method for electrostatic interactions and the Verlet integration scheme to capture atomic movements throughout the simulation timeframe. Two-dimensional interaction profiles and structural diagrams were generated using LigPlot + and Discovery Studio software.

### Mutations

Mutational analysis of peptide sequences was conducted using the MCSM platform, which applies structural modeling to anticipate stability shifts. Single-point mutations were evaluated by calculating ΔΔG magnitudes, representing changes in binding free energy between mutant and original peptides, thereby enabling identification of sequence modifications that enhance structural integrity or functional potential.

$$\Delta\Delta{G}\hspace{0.17em}=\hspace{0.17em}\Delta{G}_{original}\hspace{0.17em}-\hspace{0.17em}G_{mutant}$$

Mutations yielding positive ΔΔG magnitudes are connected with enhanced peptide stability, whereas negative magnitudes indicate destabilization. Near-zero ΔΔG readings generally reflect neutral alterations without significant structural or energetic impact.

## Results and discussion

Glycogen synthase kinase-3β represents an attractive target protein for slowing Alzheimer’s disease progression, according to its involvement in severe pathological procedures including synaptic impairment, amyloid-β accumulation, abnormal phosphorylation of tau protein, etc. Consequently, expanding the new strategies for targeting GSK-3β can pave the way for novel and efficient AD therapies. According to neuroprotective effects of existing compounds in milk^27^, in this study, the potential of κ-casein-derived peptides as candidate therapeutic molecules against GSK-3β is investigated. Various inhibitory agents against GSK-3β relevant to AD have been proposed, which are different from each other based on either their mechanisms or molecular targets. A predominant class consists of single-function agents, encompassing ATP-competitive inhibitors, non-ATP-competitive inhibitors acting at α-helical–β-sheet interfaces, and molecules suppressing enzymatic function via alternative binding sites^28–30^. The other class of inhibitors of GSK-3β are multi-target agents, which combine GSK-3β inhibition with some health advantages such as compounds mitigating oxidative stress^31^, dual inhibition of cholinesterases or BACE1^32,33^, and suppressing phosphorylation of tau protein^34^. Such multifunctional designs present a compelling approach to address the complex nature of AD pathology by modulating multiple biochemical pathways concurrently, thereby enhancing therapeutic efficacy and clinical relevance.

**Fig. 1:**
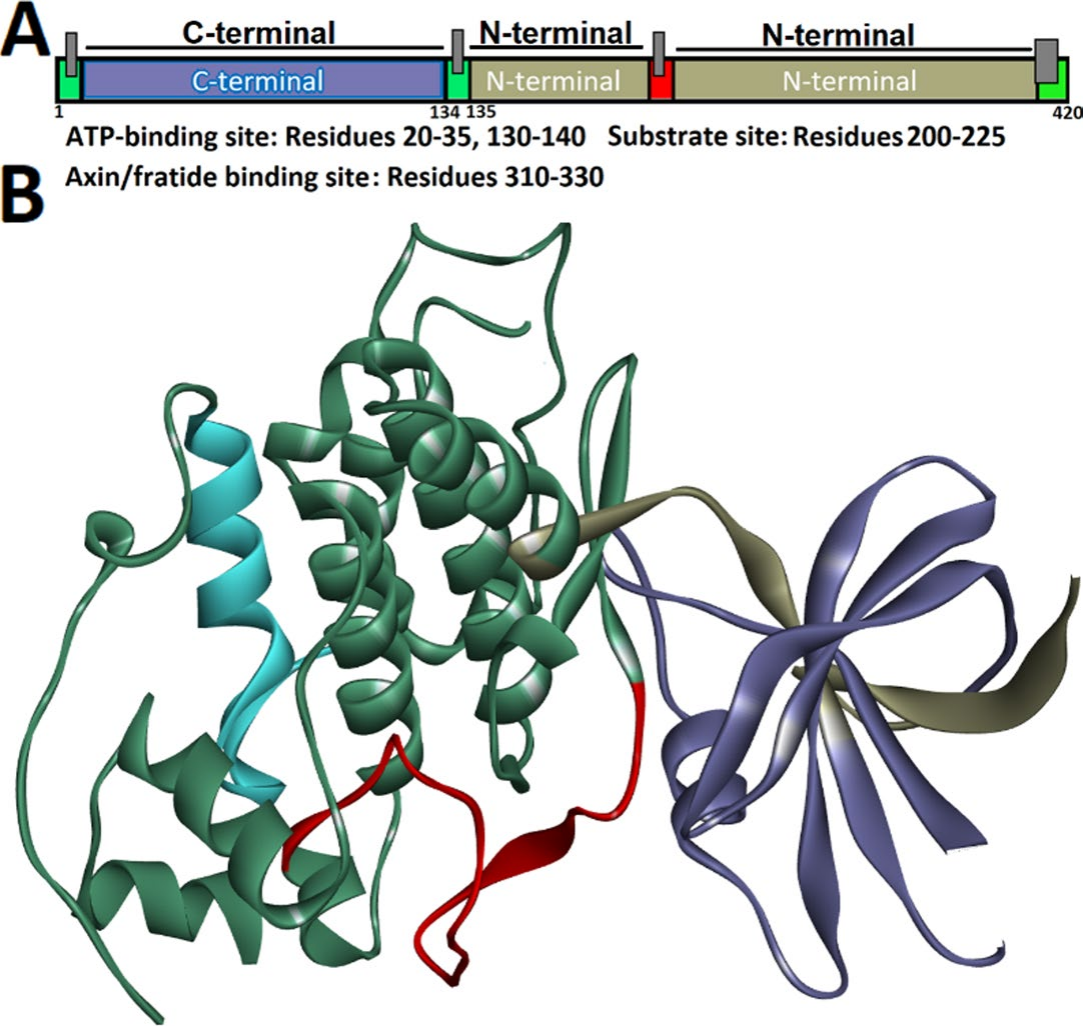
Surface visualization of GSK-3β highlighting its cavities: ATP-binding site (gray), substrate site (red), and Axin/fratide site (light blue). The GSK-3β building block includes two main terminals: a β-barrel like N-terminal, which embraces the initial 134 amino acid residues, and one C-terminal that contains the α-helices, consisting the rest of the residues. The binding site of GSK-3β embraces ATP-binding site; including residue numbers 20–35, 130–140, Substrate site; containing residues from 200–225 (this section includes the catalytic loop (Asp200), and Axin/fratide binding site; expanding from residue numbers 310 to 330.

**Fig. 2:**
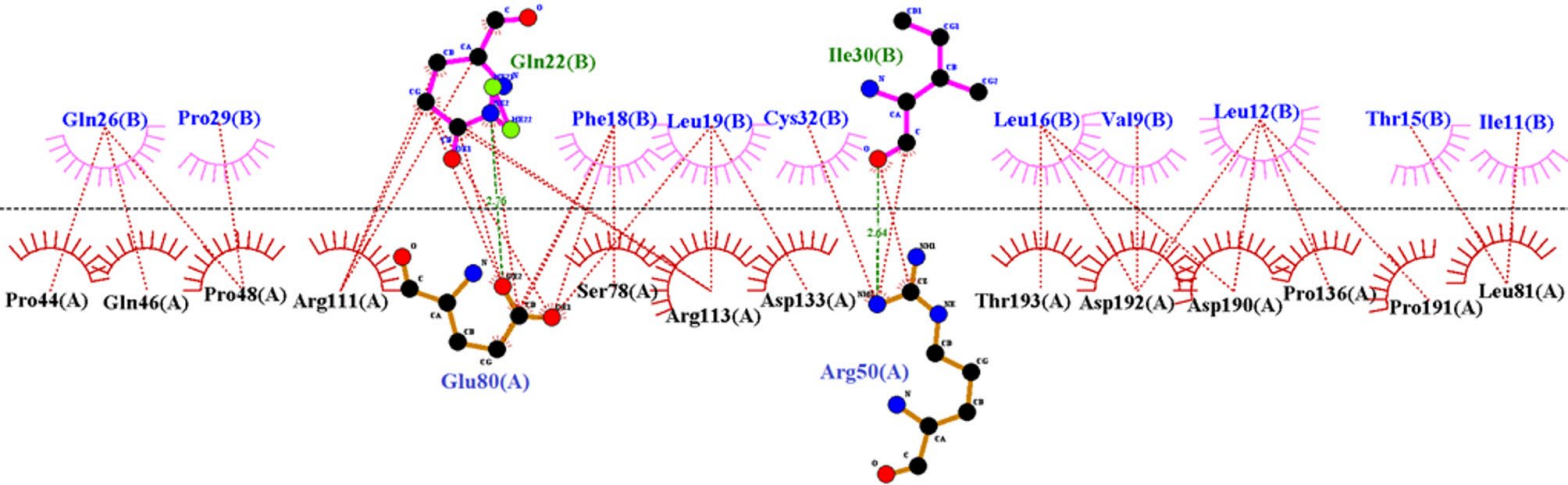
2D illustrations of the interaction between the κ-casein and the active site of GSK-3β, showing effective blockade of the binding site. The modelled κ-casein and GSK-3β structures were introduced to HADDOCK platform to identify a 10-length peptide template. Results shows that around 35 polar/nonpolar contacts were formed with residues at the binding site of the target, and importantly, around half of these interactions (nearly 17 contacts) were established by the segment 12–21 of the κ-casein, among which those nonpolar interactions with residue numbers 81, 133, 136 at the ATP-binding site of the target are significant. Thus, sequence 12–21 was chosen as the peptide template.

Structurally, GSK-3β contains two separate domains: an N-terminal comprising the initial 134 amino acids and a C-terminal region dominated by α-helices formed by the rest of the sequence. Multiple binding cavities exist, including the substrate site, ATP-binding site, Axin/fratide site, and a few allosteric pockets (Fig. 1). The current peptide inhibitor design has been motivated using 4-(4-hydroxy-3-methylphenyl)−6-phenylpyrimidin-2(5 H)-one (55E), an approved inhibitor interacting with catalytic loop (Asp200), hydrophobic pocket (Phe67, Leu132), glycine-rich loop (Gly63, Gly64, Ala83, Lys85), and the ATP-binding site (Ala83, Lys85, Asp133, Val135). This extensive interaction profile covers nearly all major functional regions with the exception of the activation loop, providing a robust structural framework for developing new GSK-3β inhibitors with improved specificity and potency (Fig. 1). As it was mentioned before, the GSK-3β and the modelled κ-casein structures were introduced to HADDOCK platform to identify a 10-length peptide template. The results have shown a binding energy of −83.7 kcal/mol. Our findings revealed that nearly 35 polar/nonpolar interactions were established with the residues located at the binding site of the GSK-3β. More importantly, around half of these contacts (~ 17 contacts) were formed by the segment 12–21 of the κ-casein, among which those nonpolar contacts with residue numbers 81, 133, 136 at the ATP-binding site of the target are significant. Thus, the sequence of 12–21 of the κ-casein was chosen as our template peptide (Fig. 2). Finally, peptide template (12–21) was extracted and subjected to 150 ns MD simulations in complex with GSK-3β, and calculate the MM/PBSA to find the residues that are prone to mutations. As can be seen in Tables 1, 2, the amino acid residues 12, 14, 15, and 18 have shown positive binding free energies, revealing that these amino acids provided limited or unfavorable share to the overall binding free energy. Actually, changing residues with unfavorable energetic profiles presents a viable approach for transforming weak molecular interactions into stronger ones, finally enhancing the cumulative binding affinity of the complex. Thus, in order to strengthen the binding affinity of the peptide template, the possible mutations to the found mutation spots (L12, L14, T15, and F18) of the peptide template were supplemented by MCSM. The MCSM platform applied ΔΔG-based analysis to introduce amino acid substitutions, quantifying binding free energy changes. Positive ΔΔG values revealed stabilizing effects.

Convincing residues at each site were combined into complete peptide variants for evaluating potential inhibitory activity against the target enzyme. Consequently, after finding and supplementations of possible mutations to the relevant sites in the peptide template, a total library of 48 peptides were generated using CHIMERA 1.8.1. In the following steps, the created library of 48 mutant peptides have been studied in terms of toxicity and allergenicity. Interestingly, nearly half of the mutant peptides (22 peptides) have passed the safety profile; including PEP1, PEP2, PEP5, PEP8, PEP9, PEP10, PEP12, PEP15,

**Table 1:** Binding free energy evaluation of the peptide template targeting GSK-3β via MMPBSA.

Residue Number	MM/PBSA energy
**12**	21.3669
13	−0.445
**14**	0.5288
**15**	2.872
16	−2.0296
17	−2.646
**18**	22.0121
19	−3.555
20	−3.272
21	−123.3

PEP18, PEP21, PEP24, PEP26, PEP27, PEP29, PEP30, PEP33, PEP36, PEP37, PEP38, PEP40, PEP44, and PEP46. Finally, these peptides have been introduced to the HADDOCK platform to uncover their inhibitory potency versus GSK-3β ATP-binding site. Armed with the docking findings, it can be concluded that four out of peptides including PEP8, PEP36, PEP40, and PEP44, demonstrated superior docking scores of −89.1, −84.6, −87.7, and − 88.4 kcal/mol, which are comparably stronger than that of the peptide template.

**Table 2:** Results of HADDOCK Docking Between Mutated Peptides and GSK-3β.

Peptide No.	PEP sequence	Docking score	VdW energy	Elec energy	Desolvation energy
**TEMPLATE**	LALTLPFLGA	**−83.7**	**−61.1**	**−348.9**	**−1.9**
PEP1	RARLLPELGA	−79.3	−45.3	−177.3	−1.9
PEP2	RAHLLPDLGA	−71.2	−33.1	−192.9	−2.9
PEP5	RARLLPDLGA	−19.4	−20.9	−52.4	5.7
**PEP8**	**H**A**HL**LP**D**LGA	**−89.1**	−57.4	−68.4	−18.9
PEP9	RARLLPHLGA	−22.2	−14.6	−45.7	−6.3
PEP10	RAHLLPELGA	−30.4	−34.9	−20.8	2.9
PEP12	HAHELPHLGA	−81.6	−39.4	−184.2	−9.8
PEP15	HARELPHLGA	−79.2	−33.8	−242.7	−0.3
PEP18	RAHELPHLGA	−78.0	−37.0	−173.8	−9.2
PEP21	RARELPHLGA	−45.9	−25.1	−113.5	−2.8
PEP24	HAHELPHLGA	−84.3	−39.1	−188.6	−9.8
PEP26	RAHDLPDLGA	−33.6	−14.9	−165.1	9.3
PEP27	HARDLPHLGA	−34.0	−24.2	−114.0	9.8
PEP29	RARDLPDLGA	−34.0	−24.2	−114.0	9.8
PEP30	RAHDLPHLGA	−39.1	−9.5	−138.9	−7.3
PEP33	RARDLPHLGA	−53.8	−28.6	−143.3	−0.8
**PEP36**	**H**A**HD**LP**H**LGA	**−84.6**	−37.2	−195.7	−9.7
PEP37	RARILPELGA	−25.0	−24.1	−44.7	5.0
PEP38	RAHILPDLGA	−78.1	−48.9	−118.3	−7.2
**PEP40**	**H**A**HI**LP**E**LGA	**−87.7**	−58.2	−59.2	−19.9
**PEP44**	**H**A**HI**LP**D**LGA	**−88.4**	−88.4	−226.6	−8.6
PEP46	RAHILPELGA	−30.6	−21.1	−114.1	6.0

It should be emphasized that the superior peptides have shown the below mutations:

- PEP8 (L12A13L14T15L16P17F18L19G20A21) ─> **H**A**HL**LP**D**LGA): L12H, L14H, T15L, F18D.
- PEP36 (L12A13L14T15L16P17F18L19G20A21)─> **H**A**HD**LP**H**LGA): L12H, L14H, T15D, F18H.
- PEP40 (L12A13L14T15L16P17F18L19G20A21)─> **H**A**HI**LP**E**LGA): L12H, L14H, T15I, F18E.
- PEP44 (L12A13L14T15L16P17F18L19G20A21)─> **H**A**HI**LP**D**LGA): L12H, L14H, T15I, F18D.

It is noteworthy mentioning that all of the chosen peptides have L12H, L14H replacements in common, demonstrating the substitution of leucine with histidine. This in turn shows that the replacement of a moderately hydrophobic, smaller and polar amino acid near the peptide’s N-terminus might improve its ability to bind. Interestingly, the substitution of F18 with an aspartic acid, as observed in PEP8 and PEP44, leads to the enhancement of binding affinity since an exchange of a hydrophobic side chain is replaced by a negatively charged one, and significantly strengthens their binding affinities. However, substitution of the phenylalanine (F18) with either histidine or glutamic acid does not impose a remarkable improvement in binding affinity, as appeared for PEP36 and PEP40.

Surprisingly, exchanging of threonine (L15) either with leucine or Isoleucine leads to an important enhancement of binding affinity. The phenomena can refer to the higher hydrophobic nature of leucine/Isoleucine in comparison to threonine, resulting in stronger hydrophobic (Van der Waals) contacts, improving binding strength at the binding cavity of the GSK-3β. This interpretation can be related to higher binding scores of PEP8 and PEP44. However, this explanation is covered by the fact that when this L15 is replaced by an Asp; a smaller and negatively charged residue (like PEP36), the binding affinity becomes weaker since the substitution of a polar residue at this position can diminish the binding strength of the mutant peptide.

**Fig. 3:**
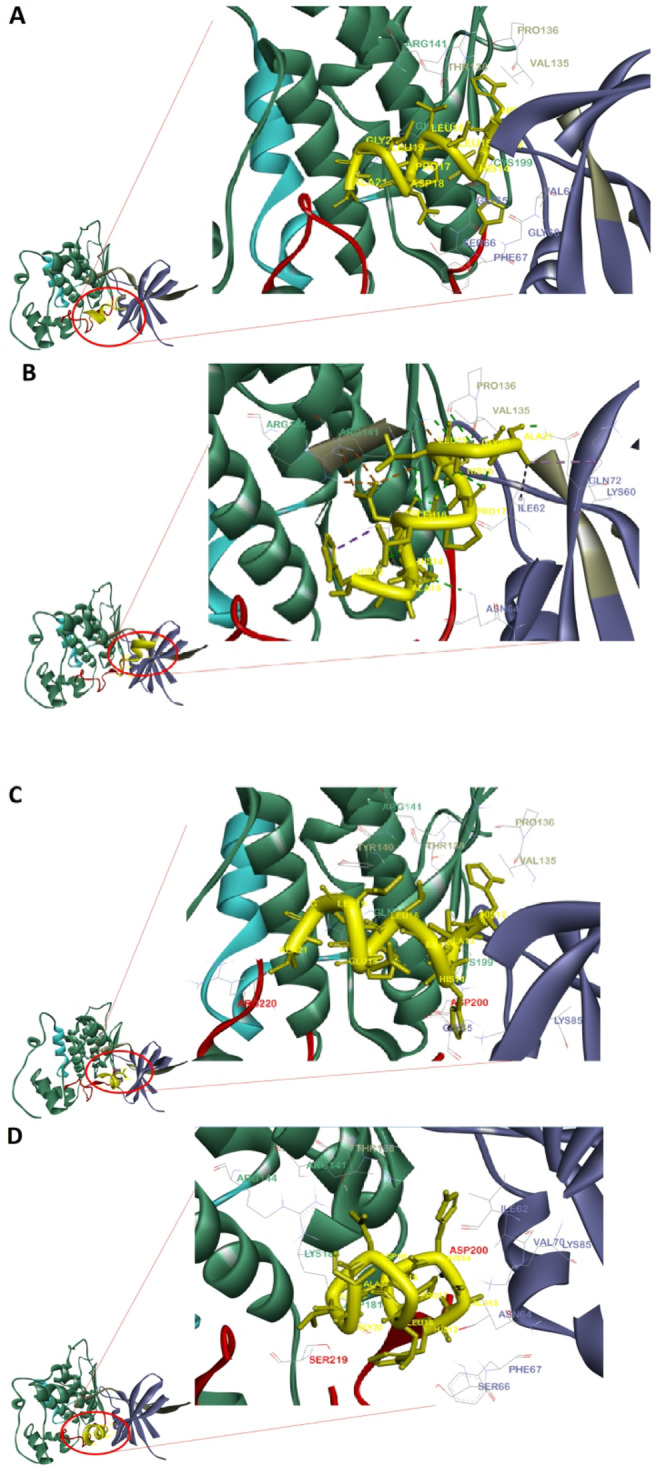
3D representations of (**A**) PEP8, (**B**) PEP36, (**C**) PEP40, and (**D**) PEP44 with the binding site of the GSK-3β.

**Fig. 4:**
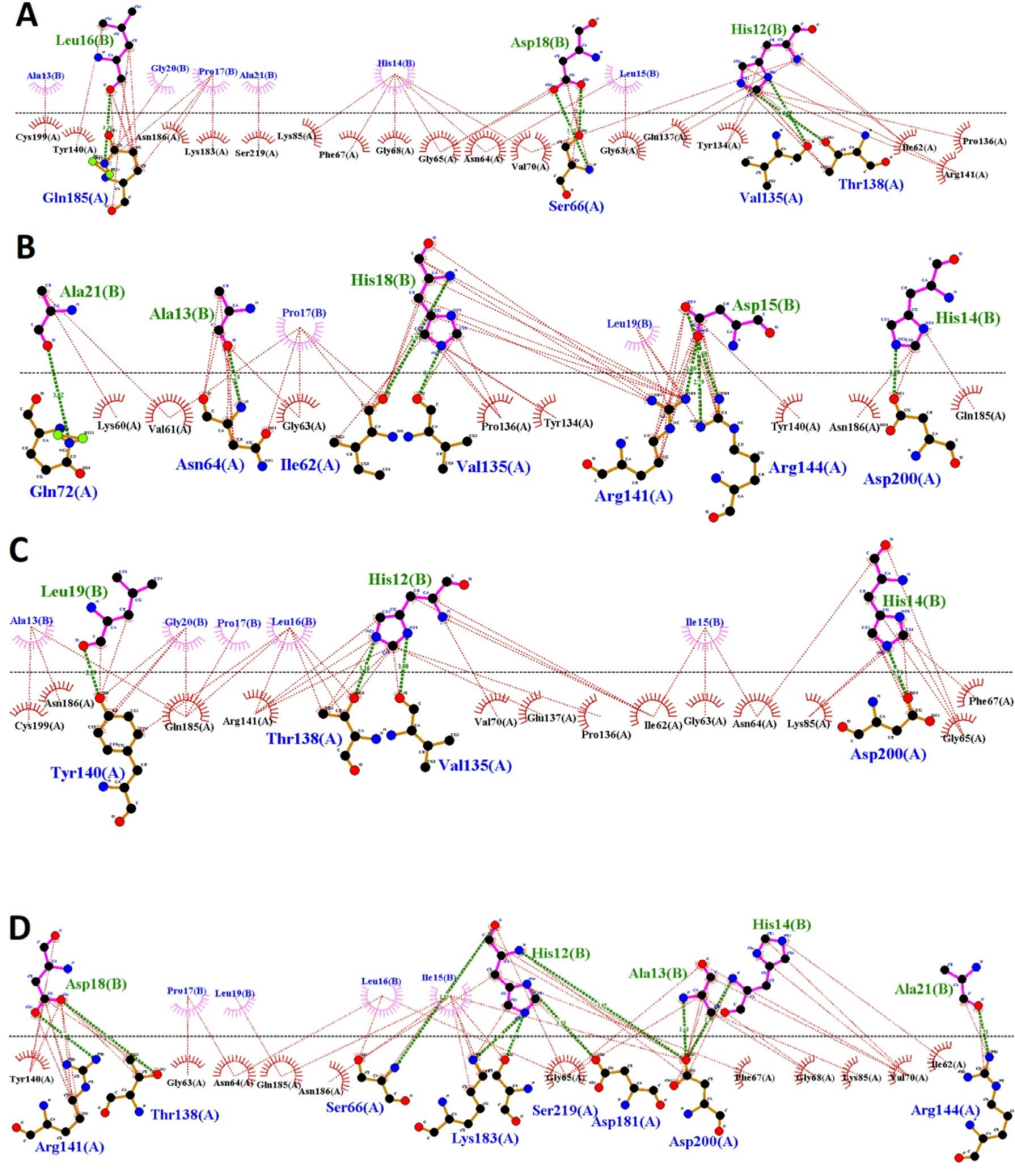
2D exhibitions of (**A**) PEP8, (**B**) PEP36, (**C**) PEP40, and (**D**) PEP44 with the cavity site of the GSK-3β. All of the mutant peptides somehow established contacts with the glycine-rich loop residues such as Gly63, Gly65, and Lys85. As we know, glycine-rich loop shares two same Asp83 and Asp85 residues with the ATP-binding site^24^. This means that these peptides in general bind to glycine-rich loop at the ATP-binding site, which is normal since half of their sequences are similar. More importantly, all of the four mutant peptides, except for PEP8, made hydrogen bonds with the catalytic loop-Asp200, highlighting their inhibitory potential against GSK-3β enzymatic actions. The only differences between their binding paradigms are types of interactions, the residue types, and engagement with lower/larger number of residues located at the active site of the GSK-3β (see page 15 and Table 3 for more details).

According to the 2D exhibitions of the chosen peptide with the target protein (Figs. 3, 4), it can be stressed that PEP44 and PEP8 demonstrated stronger binding affinities to the GSK-3β binding pocket. Although the former has established two times greater number of polar interactions than that of the latter; ten and five, higher number of polar contacts could not guarantee its binding affinity since PEP8’s binding strength is governed by higher number of contacts; in total this peptide shows interactions with 23 amino acid residues, and warrantees the superiority of its binding affinity. Surprisingly, the greatness of contact points also organize the binding affinity of the other peptides since the binding affinities follow the order of PEP8 > PEP44 > PEP40 > PEP36, and interestingly, the number of contacts of the PEP8, PEP44, PEP40, and PEP36 with the target residues stand for 21, 19, 17, and 15, respectively. These findings clarify that a larger network of interaction sites appears to strengthen overall affinity, even if each one offers only a small energetic contribution on its own. In addition, the involvement of more residues in interaction increases the specificity, allowing the inhibitor to be more effectively distinguished from competing ligands or natural substrates, thereby improving selectivity. The target-peptide complexes possessing a larger network of interaction sites are likely to align more accurately within the ATP-binding site.

As can be observed in Table 3, two superior peptides, PEP8 and PEP44, in terms of binding affinity have illustrated similar binding paradigm with few differences, e.g. two peptides get engaged with the glycine-rich loop and ATP-binding pocket of the target. However, PEP8 also shows contacts with few residues (like Tyr134, Val135 (1), Pro136, Glu137, Thr138 (1), Tyr140, Arg141) near the ATP-binding pocket, and Cys199, while PEP44 contacted with the catalytic residue Ap200, Asp181, and Arg44. This phenomenon can propose minor differences in their binding modes. The other two peptides (PEP40 and PEP36) somehow demonstrated similar binding modes in the GSK-3β active pocket with lower number of contacts. It is interesting to note that all of the four mutated peptides have indicated at least one/two polar interactions with the residues like Val135, Thr138, and Lys85 located at the ATP-binding pocket, especially PEP8 with Val135, Thr138, highlighting their potential to make some serious disturbances in the exact binding of the substrate to the active site of the kinase, malfunctioning its enzymatic activities.

**Table 3:** The table outlines residue contacts formed between the peptides and GSK-3β. Values shown in parentheses correspond to hydrogen bonds, whereas the other listed residues represent hydrophobic interactions.

Peptide No.	The target amino acids
PEP8	Ile62, Gly63, Asn64, Gly65, Ser66 (2), Phe67, Gly68, Val70, **Lys85**, Tyr134, **Val135** (1), Pro136, Glu137, **Thr138** (1), Tyr140, Arg141, Lys183, Gln185 (1), Asn186, Cys199, Ser219
PEP36	Lys60, Val61, Ile62 (1), Gly63, Asn64 (1), Gln72 (1), Tyr134, **Val135** (1), Pro136, Tyr140, Arg141 (1), Arg144 (2), Gln185, Asn186, **Asp200** (1)
PEP40	Ile62, Gly63, Asn64, Gly65, Phe67, Val70, **Lys85**, **Val135** (1), Pro136, Glu137, **Thr138** (1), Tyr140 (1), Arg141, Gln185, Asn186, Cys199, **Asp200** (1)
PEP44	Ile62, Gly63, Asn64, Gly65, Ser66 (1), Phe67, Gly68, Val70, **Lys85**, **Thr138** (1), Tyr140, Arg141 (1), Arg144 (1), Asp181 (1), Lys183 (1), Gln185, Asn186, **Asp200 (3)**, Ser219 (1)

**Fig. 5:**
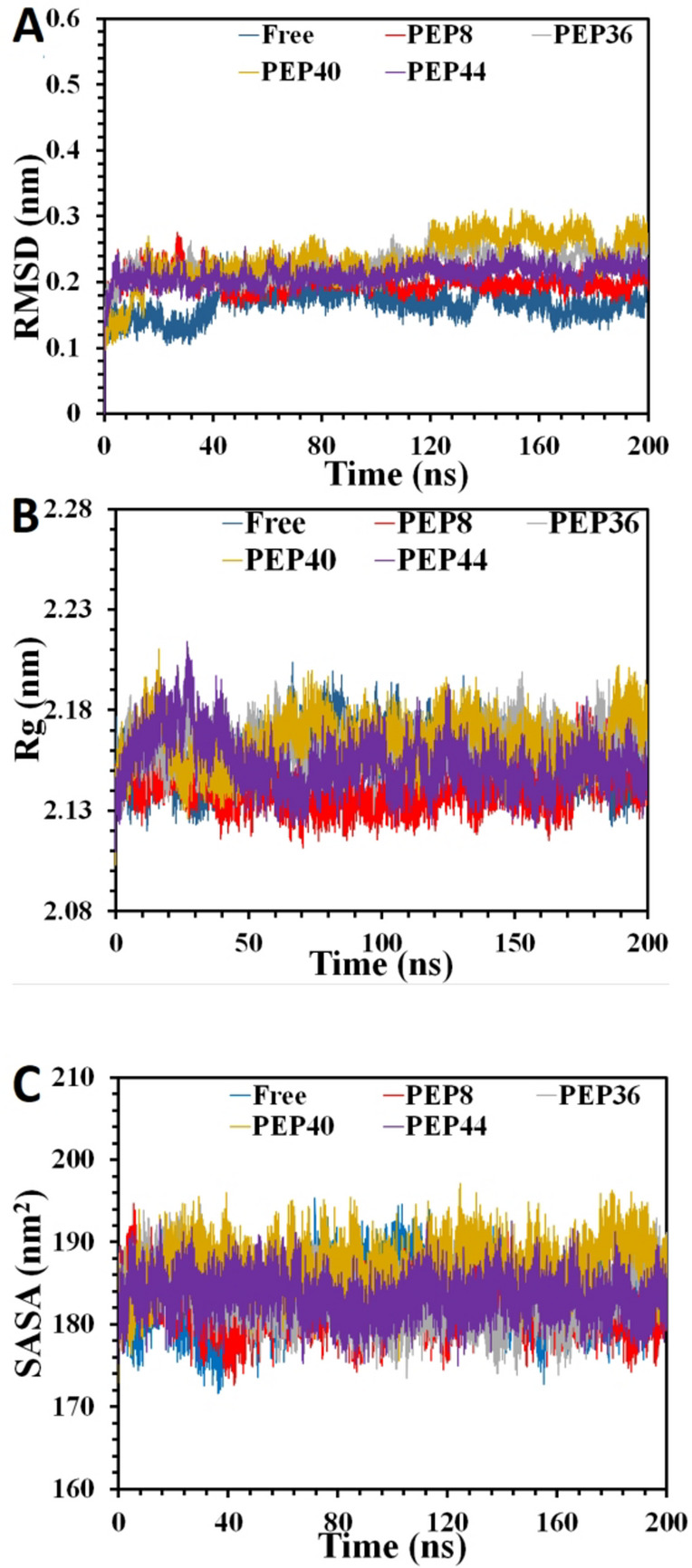
The (**A)** RMSD, (**B)** Rg, and (**C)** SASA plots of the free GSK-3β (blue) and its complex with PEP8 (red), PEP36 (gray), PEP40 (dark orange), and PEP44 (purple).

In order to uncover the contact nature of the designed peptides on the dynamics of the target, 200 ns MD simulations have been fulfilled including the apo-GSK-3β and in complex with the four peptides. As is illustrated in the Fig. 5A, the RMSD diagram for the apo-GSK-3β undergoes a medium uprising over the starting 60 ns with the RMSD magnitude of ~ 0.21 nm, which might be due to relaxation state, before very smooth reductions to get plateaued after around 70 ns along with few small fluctuations over the simulation period, with mean magnitude of ~ 0.099 nm. These phenomena reveal that the apo-protein is not speculated to go through harsh fluctuations and experience little structural changes through the era of 200 ns simulation time, postulating that this system may reach its equilibration form and this simulation time is anticipated to be enough for such protein.

Remarkably, among the four mutated peptides, complexes consisting of PEP8, PEP44, and somehow PEP36 are predicted to have lesser fluctuations in their RMSD plots. This refers to the matter that the binding of these peptide sequences, particularly PEP8 and PEP44, is likely to bring about some rigidity in the overall structure of the target enzyme and decrease its conformational freedom, leading to important structural limitations. However, the RMSD plot for the system comprising PEP36 demonstrates higher fluctuations although the RMSD plot for the complex containing PEP40 reveals higher magnitudes of fluctuations. This means that the protein might have higher flexibility upon binding to these two peptides, particularly upon binding to PEP40, leading to formation of weaker complexes in comparison to the systems comprising PEP8 and PEP44. Overall, among all, PEP44 and PEP8 cause more limitations in the target structure, while the binding of PEP8 introduces more restrictions in the GSK-3β domains, and forms a more limited conformation and stable complex with the target enzyme than the other complexes.

It is interesting to note that the average RMSD values for the peptides, especially PEP8 and PEP44, can support our interpretations since the mean RMSD magnitudes for free protein stands for 0.099 nm (free protein), 0.101 nm (PEP8), 0.106 nm (PEP36), 0.129 nm (PEP40), and 0.110 nm (PEP44), correspondingly. Besides, the Rg analysis, as an important tool for any possible changes in the protein dimension by calculation of a protein atoms’ distribution around the protein center of mass, for the free protein and in the presence of the four peptides (Fig. 5B). The Rg plot of the protein in complex with PEP8 and PEP44 do not show any specific changes disregarding of few small rising and reductions, showing slight opening and closure in the protein structure upon binding to these two sequences, while with the slightly lower mean Rg magnitudes of 2.120 nm and 2.122 nm compared to that of the free protein; 2.128 nm. However, the more extended conformation can be seen in the systems comprising PEP40 and to some point PEP36 in comparison to the apo-enzyme, with the mean Rg values of 2.138 and 2.131, respectively. According to these data, it should be stressed that the target do not exhibit any specific unfolding phenomenon, while the constant/slightly lower Rg values under binding to PEP8 and PEP44 may highlight moderately higher compactness in the target structure.

Moreover, the SASA analysis for all complexes (Fig. 5C), except for complex with PEP40, revealed major and moderate reductions from 179.95 nm^2^ (free protein) to 177.18 nm^2^ (PEP8), 178.56 nm^2^ (PEP44), and 179.04 nm^2^ (PEP36), respectively. The average SASA values for the system containing PEP40 also stand for 181.73 nm^2^. These results clarify the possible compression in the conformation of the GSK-3Β, and the most compressions occurs upon binding to PEP8 and PEP44, which the phenomena can decline the exposing of the protein to the solvent. This means that binding of these two peptides introduce a tighter packing to the target conformation, which this compression can prevent the access of protein’s hydrophobic regions to water exposure, limiting the freedom movement of vital loops approximate to the active pocket, finally hindering substrate access/binding and malfunctioning the enzymatic duties. In comparison to complexes consisting PEP8 and PEP44, however, apo-protein has a higher solvent accessible surface area (SASA average of 179.95 nm^2^), allowing it to be more exposed of solvent, enhancing substrate access and catalytic functions.

**Fig. 6:**
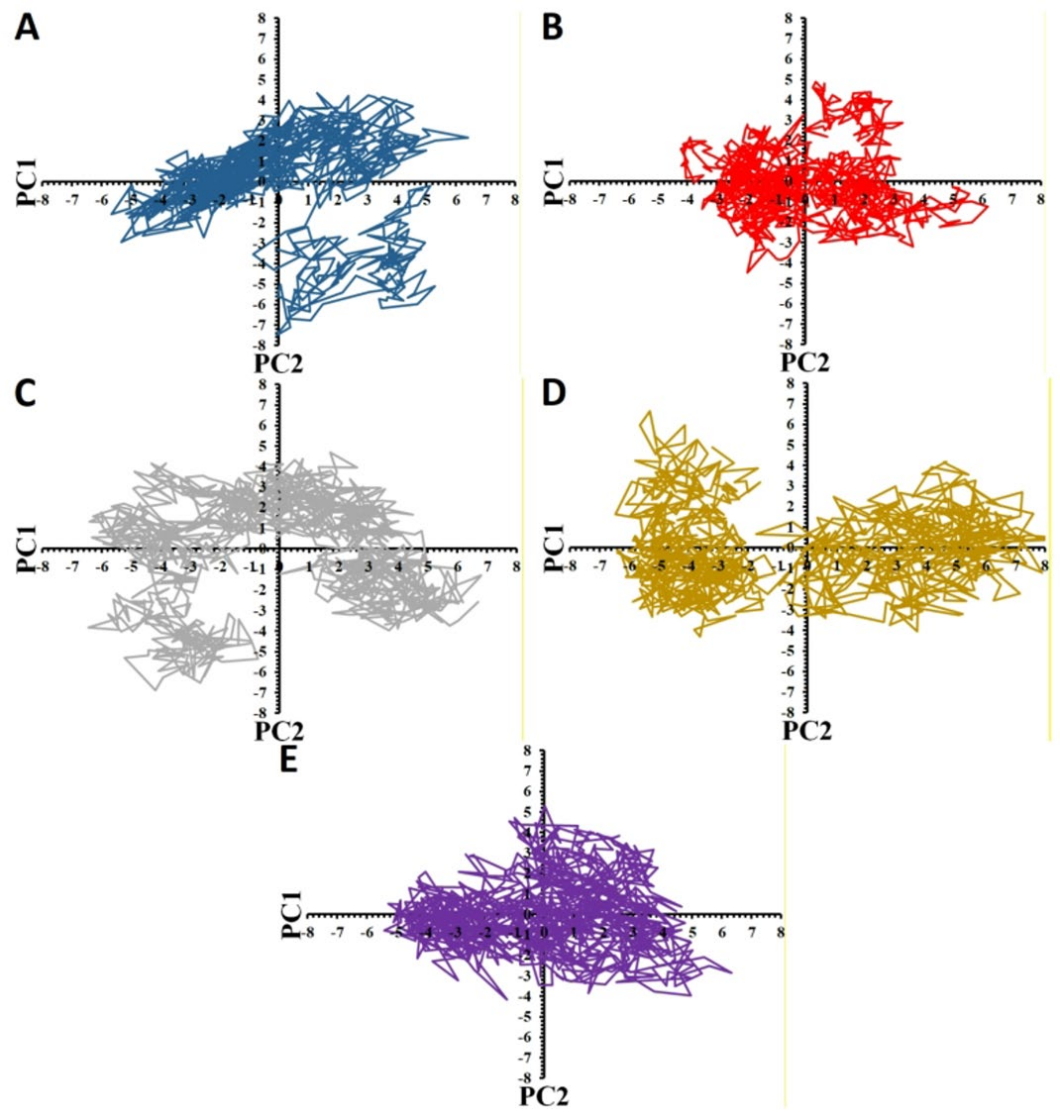
Presentation of the PCA protein dynamics, illustrating the conformational behavior in (**A**) the ligand-free and in complex with (**B**) PEP8 (**B**), (**C**) PEP36, (**D**) PEP40, and (**E**) PEP44, extracted from the final 10 ns (1000 frames) of the simulation trajectories.

PCA was applied to the last 10 ns of every trajectory to explore conformational alterations and dynamic behavior of the target protein in both its ligand-free and complexed forms. The target protein has indicated two separate and expanded clusters, revealing the range of structural conformations it adopted during the simulation. This scale of dispersion highlights considerable freedom of move owing to the expanded spread observed (Fig. 6A). The PCA range of the free protein expands from − 6 to 7 on PC1 (range = 13) and − 7.5 to 4.5 (on PC2; range = 12). However, the GSK-3β in complex with the mutant peptides exhibits distinguishable mobility paradigm and the range of protein movements is anticipated to face some changes in particular in the complex with PEP8, PEP44, and PEP36. This observation may refer to different conformational states that will be adjusted by the protein in the proximity of the peptides, and importantly, the tighter clustering observed for the peptide-target complexes reveals restricted structural variations, highlighting that these systems occupy a more confined conformational landscape (Figs. 6B-D). For example, the PCA movement paradigm is seen to face some changes, particularly in the complexes with PEP8, PEP44, and PEP36, while the most restrictions in the mobility ranges of the target take place after binding to PEP8 and PEP44. The PCA plot of the PEP8-GCK-3β system has demonstrated the most limited motion range across the initial two PCs, ranging from − 4 to 6 on PC1 (range = 10) and between − 4.5 and 5 on PC2 (range = 9.5). As for the other two complexes with PEP36 and PEP44, the former complex shows somehow more deformity in the protein motion pattern, while the latter complex possesses the higher degree of restrictions in the protein movement range since the mobility range varies between − 5.4 and 6.5 (PC1; range = 11.9) and from − 4.5 to 5.4 (PC2; range = 9.9). Comparatively, PEP36 introduces a bit larger movement to the GSK-3β structure since the movement range of the target varies from − 6.5 to 6.5 and − 6.6 to 4.5 on the first two PCs, with the motion ranges of 13 and 11.1 on PC1 and PC2, respectively. These observations demonstrate that both peptides change the protein motion paradigm by inducing different degrees of freedom in the protein conformation. PEP40 could also change the motion pattern of the protein, while with the least restrictions in the mobility range since the motion range of the target after binding to the peptide alter between − 6.5 and 7.7 (on PC1; range = 14.2) and from − 4 to 7 (on PC2; range = 11), suggesting that the GSK-3β experiences a higher degree of freedom upon binding to PEP40 with no sign of noticeable unfolding phenomenon, which is in the same line with the Rg results.

### Total explanations of RMSD, Rg, SASA, PCA results of the studied complexes

The RMSD analysis revealed that PEP8 and PEP44 have shown some limitations in the protein structure. Thus, it was followed by chasing any possible changes in the protein size and structure by Rg analysis. The Rg results have approved these limitations in the protein structure upon binding to PEP8 and PEP44 since the mean Rg values of the protein in complex with these two peptides shows slight reductions. However, as the RMSD analysis indicated small increases in the RMSD average of the protein in complex with PEP36 and PEP40, the mean Rg values of the protein with these two peptides have also escalated marginally.

So, limitations/compactions in the protein structure upon binding PEP8 and PEP44, and slightly more extended conformation after binding to PEP40, and to some point upon binding to PEP36, are shown by Rg. To further investigation of these phenomena, SASA analysis was followed. The SASA results indicated that the average SASA values of free protein shows decreases upon binding to PEP8, PEP44, and PEP36, respectively, while the average SASA magnitudes for the system containing PEP40 delineated a slight increase. These results are in line with those of the Rg results and approved the slight compression in the protein structure after binding to PEP8, PEP44, and a margin more extended conformation after binding to PEP36.

In order to more seeking these compression/extended conformation of the protein upon binding to these peptides, PCA was followed. Interestingly, PCA results have approved the slight compression in the protein conformation upon binding to PEP8 and PEP44 since the mobility range of free protein on PC1 and PC2 are 13 and 12, and after binding to PEP8 and PEP44 these ranges decreased to 10 and 9.5 (for PEP8) and to 11.9 and 9.9 (for PEP44). These results are in accordance with the Rg/SASA results, and have confirmed compression in the protein structure. PEP36 also induced slight compression into the protein mobility range to 13 and 11.1 on the initial two PCS, which agrees the Rg and SASA results. Fascinatingly, marginally extended conformation upon binding to PEP40, as observed in SASA and Rg analysis, was also approved in the PCA results since this peptide caused a slight increase in the movement pattern of the protein to 14.2 on PC1.

Taken these together, in total, it can be concluded that the highest degrees of the decrease in the mobility range of protein take place in the order of PEP8 > PEP44 > PEP36 > PEP40 on the initial two PCs in comparison to the protein in the free state. This interpretation means that the structure of the protein undergoes a decreased/limited configurational freedom upon binding to the peptides, which this decrease in the protein flexibility can constrain its capacity to find different range of configuration. In its apo state, the protein shows an expanded range of displacement along the initial two PCs, which usually represents the dynamic nature required to support enzymatic function, encompassing substrate recognition, regulatory allosteric changes, etc. However, in complex with these peptides, the configurational freedom and structural flexibility of GSK-3β seem to be limited after binding to these peptides, especially upon binding to PEP8 and PEP44, resulting in a more compact/restricted and possibly less effective enzymatic condition.

**Fig. 7:**
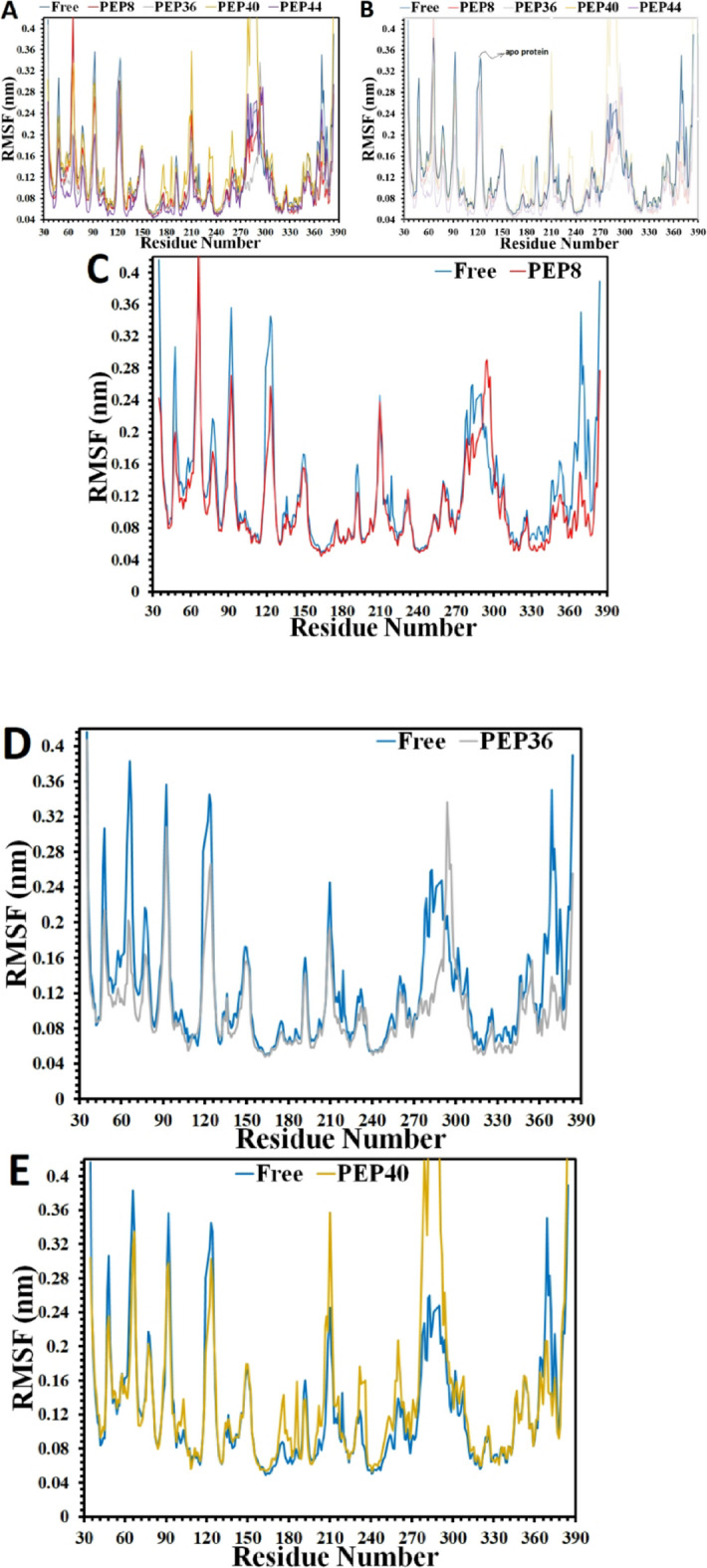

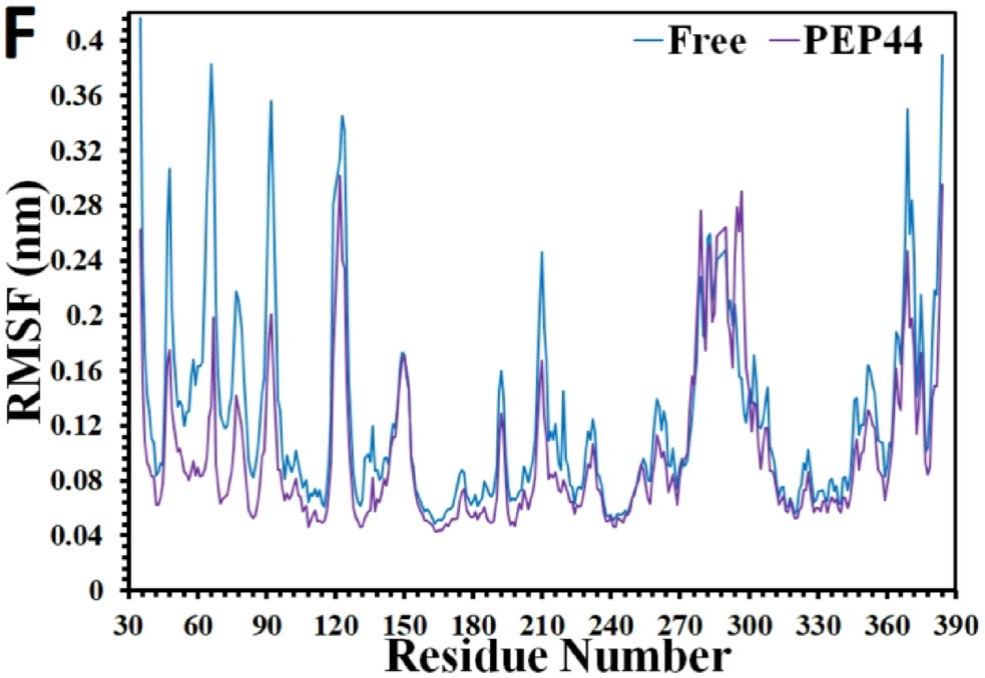
Changes in the RMSF analysis of apo protein (blue) **(A-B)**, and in complexes with (**C)** the PEP8, **(D)** PEP36, (**E)** PEP40, and (**F)** PEP44. (the apo protein and in the complexes with the peptides are shown in Fig. 6A-B, while the separated complexes of the peptides-protein systems are exhibited in Figs. 6C-F, showing general reductions in the RMSF fluctuations of the protein residues upon binding to the peptides).

Armed with the PCA analysis that denote conformational dynamics leading to entropic contribution, in this line, more conformational dynamicity and fluctuations of the protein’s residues atoms are explored by means of RMSF analysis. As can be detected in the Fig. 7A-B, in general, in comparison to the RMSF fluctuations of the residues of the apo-protein, these numbers demonstrate lower oscillations almost in all complexes, except for the complex containing PEP40 which shows almost higher RMSF magnitudes in few parts of the protein. Remarkably, these findings are in cooperate with the previous results, confirming that the protein is anticipated to face decreased mobility, and experience some inflexibilities upon binding to the peptides, in particular after binding to PEP8 and PEP44. These findings may highlight the larger freedom degree of the apo-protein, while restricted mobility upon peptide binding.

In order to seek how the RMSF values of these residues change upon binding to the mutant peptides, the RMSF diagram of each complexes are shown in proximity to that of the apo-GSK3-β in Fig. 7C-F. In the complex comprising PEP8, some reductions are observed in the RMSF oscillations of residue numbers around 34–36, 47, 55–60, 71–76, 83–85 (the glycine-rich loop, ATP-binding site), 133–136 (ATP-binding site), 93, 103, 119–125, located at the N-terminal 144, 149, 160, 172, 192–195, 199, 210, 213–220, 267, 279–286, 308, 315 (Axin/fratide site), 348–356, and 366–376 situated at the C-terminal. However, the RMSF fluctuations are seen to rise in few spots near the residue numbers 66, and 292–298. Almost the same pattern is predicted in the RMSF diagram of the system comprising PEP44 since the RMSF numbers nearly show significant drops in the C- and N-terminal domains of the GSK-3β, e.g. these numbers demonstrate some declines near amino acid residues 37–44, 49–60, 63–67 (the glycine-rich loop), 80–85 (ATP-binding site), 92–94, 99–115, 123–125, 132–135 (ATP-binding site), 136 (situated at the N-terminal), 168–171, 183–185, 193, 200–203 (catalytic loop), 213–220, 259–264, 303–307, 309–316 (Axin/fratide site), 346, and 352 at the C-terminal domain. As for the complex with the PEP36, the RMSF fluctuations seem to experience the same paradigm, but with lower degrees of reductions, compared to the systems containing PEP8 and PEP44. For example, these numbers are anticipated to drop around residue numbers 47, 65–68, 71–75, 82, 90–95, 111–113, 133, 136 (ATP-binding site), 141–149, 176, 186, 192, 199–201 (catalytic loop), 210, 218–220, 232, 260–262, 251–255, 276–291, 309–324 (Axin/fratide site), 352, and 364–376 located at the C-terminal. However, the RMSF fluctuations undergo some risings approximately around residues located at the C-terminal and few spots situated at the N-terminal domain of the protein upon binding to PEP40, e.g. these numbers increase near residue numbers 42–45, 53, 58, 101–104, 113 (at the N-terminal domain), and more spots located at the C-terminal such as residue numbers 149, 166–179, 184–187, 199–203 (catalytic site), 210–212, 230–237, 249–256, 258, 262, 283–293, 308–311, 326, and 353. Nonetheless, few spots’ RMSF fluctuations diminish around residue numbers 47, 66, 92, 123 (at the N-terminal domain), 192, 219, and 365–373 (at the C-terminal domain).

Armed with the RMSF results, it should be highlighted that peptide binding generally reduced the flexibility of GSK-3β compared to the apo form, with notable reducing trend around residues located at the ATP-binding site upon binding in the order of PEP8 > PEP44 > PEP36, which these results are in line accordance with the PCA results, showing the entropic contribution that is caused by conformational dynamics upon peptide binding. Besides, PEP8 and PEP44 exerted the strongest effects, inducing pronounced reductions in residue fluctuations across N- and C-terminal domains. These limitations cooperate with the Rg and SASA results, supporting the hypothesis of limitations and inflexibilities/rigidity in the protein structure upon binding to these peptides, leading to induce important negative impacts on the enzymatic functions of the target enzyme. In contrast, PEP40 displayed localized increases in mobility, indicating weaker stabilizing capacity relative to other peptides.

**Fig. 8:**
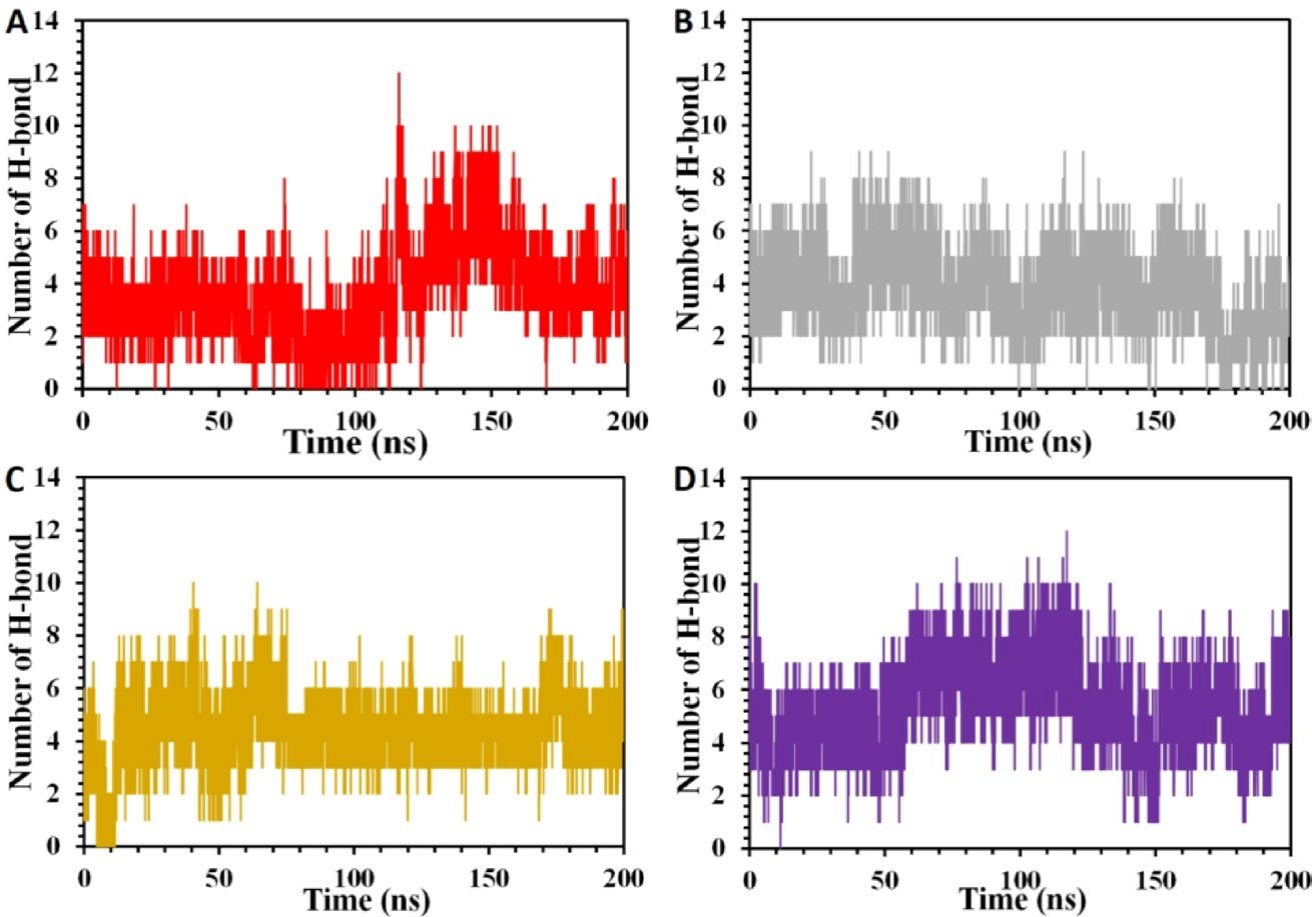
H-bond analysis of the GSK-3β in complex with (**A)** PEP8, (**B)** PEP36, (**C)** PEP40, and (**D)** PEP44.

Furthermore, the pattern of H-bonding between the mutant peptides and the protein is investigated (Fig. 8A-D), showing a considerable dynamism throughout the simulation. As it was observed in the molecular docking results, the order of the hydrogen bonds between the peptides and the protein follows the order of PEP44 > PEP36 > PEP8 > PEP40. After MD simulations, the hydrogen binding pattern almost follow the similar pattern with some differences, which goes following order of PEP44 > PEP8 ~ PEP40 > PEP36, and varies in terms of the number of polar interactions made in each complexes. For example, like molecular docking results, PEP44 and PEP8 have established higher hydrogen bonds among all, forming six and five hydrogen bonds over the simulation period. Unlike the docking results, MD simulations results have revealed higher polar contacts for the complex containing PEP40 (five stable hydrogen bonds) in comparison to those made by PEP36. By contrast, PEP36-protein complex is seen to have four stable H-bonds until the end of simulation time. Apart from these results, MD findings also exhibit that PEP8 and PEP44 complexes’ H-bindings were stable through the simulation with few fluctuations around the middle of the simulation period. The other complex comprising PEP36 showed almost lower polar contacts and somehow unstable hydrogen bonds, while the system comprising higher H-binding with smaller fluctuations. Higher number of hydrogen bonds, more strong and stable complex will become, thus, PEP44 and PEP8 are predicted to make stronger complexes with the target protein.

As the effect of peptide binding on the protein conformational dynamics was assessed by PCA and RMSF analysis in terms of entropic contribution, the enthalpic contribution of the phenomena can also be investigated by calculation of the total binding energies of each studied peptides in complex with the protein through MMPBSA analysis (Table 4). Interestingly, the MMPBSA calculations illustrate that PEP44 and PEP8 have established the strongest complexes with the GSK-3β with the total binding energies of −188.482 kJ/mol and − 184.404 kJ/mol, followed by PEP36 (−121.938 kJ/mol) and PEP40 (−86.954 kJ/mol). It is noteworthy to mention that almost van der Waals forces possess the biggest share in the total binding energies of each complexes, with PEP44 and PEP8 have shown superior VdW energies, demonstrating these two peptides are predicted to establish strong hydrophobic contacts with the target ATP-binding site. The biggest shares of electrostatic energies can be seen in the complex with PEP36 (−292.135 kJ/mol), followed by PEP44 (−166.259 kJ/mol) and PEP8 (−89.358 kJ/mol). This shows that PEP36 and PEP44 form the most pronounced electrostatic contacts with the target protein, while the former the highest polar solvation contribution, causing its total binding energy to become smaller in comparison to the other two peptides (PEP44 and PEP8), indicating a stronger desolvation cost, typically associated with highly polar or ionized contacts. The high polar solvation energy of the complex containing PEP36 perhaps lies in its conformational elements. For example, the positioning of its polar/charged residues like Asp15 or His18 relative to the solvent channel. Among the peptides, PEP8 shows the most promising SASA energy, implying that it conceals more surface region during complex formation than the others. PEP40 also forms the weakest complex with the target protein due to its lower total binding energy of −86.954 kJ/mol, which its complex is basically governed by hydrophobic contacts. To sum, PEP44 and PEP8 have established the most strengthen complex with the GSK-3β due to their higher VdW and electrostatic energies, highlighting that these two peptides can be strong binders among all studied peptides. PEP36 also forms a strong complex with the protein, while its total bonding energy is weaker owing to its higher polar solvation energy.

One point should be highlighted here; ATP-binding sites are structurally similar among different kinases, and one of the most significant challenges in kinase inhibitor discovery is to identify selective inhibitors^35^. Interestingly, in general, our results suggest that the κ-casein-derived peptides may achieve some specificity through extended and multivalent interaction networks beyond canonical ATP anchoring residues. Unlike small-molecule ATP mimetics, PEP8 and PEP44 establish contacts not only with the ATP-binding residues (e.g., Lys85, Val135, Asp133), but also with the catalytic loop (Asp200). Molecular dynamics simulations further demonstrate that peptide binding induces localized compaction and restricts conformational freedom or mobility movement of GSK-3β, particularly around the glycine-rich loop and the catalytic region. These interactions might hinder the catalytic activities of GSK-3β. Thus, the relatively larger size, sequence complexity, and casein-derived scaffold of these peptides could confer a higher degree of functional selectivity compared to classical small-molecule ATP-competitive inhibitors, strengthening their therapeutic relevance.

**Table 4:** Mean MM/PBSA interaction energies for the mutant peptide in complex with GSK-3β (kJ/mol).

Peptide No.	VdW	Elec	Polar solvation	SASA	Total
PEP8	−157.772	−89.358	80.422	−17.652	**−184.404**
PEP36	−143.168	−292.135	321.354	−7.989	−121.938
PEP40	−67.746	−33.113	29.797	−15.892	−86.954
PEP44	−163.653	−166.259	149.625	−8.195	**−188.482**

## Conclusion

GSK-3β is a compelling therapeutic target in Alzheimer’s disease due to its central role in tau hyperphosphorylation, amyloidopathy, and synaptic dysfunction. Armed with the neuroproteiction of milk proteins, in the given study, a κ-casein-derived (L12A13L14T15L16P17F18L19G20A21)) template was identified through HADDOCK (− 83.7 kcal·mol). The peptide template (L12A13L14T15L16P17F18L19G20A21) was then introduced to MD simulations to find the spots that are prone to mutations using the calculation of the MM/PBSA of each residue, revealing four mutations sites provided unfavorable share to the overall binding free energy. To enhance peptide binding affinity, we used MCSM to rationally mutate energetically unfavorable positions (L12, L14, T15, and F18) using ΔΔG-based substitution analysis and generated a library of 48 peptides. Of which, 22 passed toxicity/allergenicity filters. Subsequent docking and 200-ns MD workflows prioritized four leads—PEP8, PEP36, PEP40, and PEP44—with PEP8 (− 89.1 kcal·mol⁻¹) and PEP44 (− 88.4 kcal·mol⁻¹) emerging as top candidates. The 22 mutant peptides was subjected to HADDOCK server, and identified four peptides (PEP8, PEP36, PEP40, PEP44) with superior binding scores (−89.1 to −84.6 kcal/mol) over the template. The four mutant peptides shared L12H/L14H mutations, while F18→Asp (PEP8, PEP44) and T15→Leu/Ile could enhance binding affinity. PEP8 showed the strongest binding strength, forming 21 contacts, followed by PEP44, PEP40, and PEP36, with 19, 17, and 15 contacts, respectively. These findings highlighted that larger interaction networks can lead to stronger binding and selectivity. PEP8 specifically contacted with the residues at the ATP-binding pocket (Val135, Thr138, Tyr134), while PEP44 contacted catalytic Asp200. Overall, PEP8 and PEP44 emerged as the most promising inhibitors, disrupting substrate recognition at the GSK3-β ATP-binding site.

The MD results were investigated in terms of entropic and enthalpic contributions to assess the effect of peptide binding on the protein dynamics. MD results revealed some reductions in RMSD fluctuations, which was traced by slightly lower Rg and SASA average values, showing slight compression after binding to PEP8, PEP44, and PEP36. The entropic contribution was assessed by PCA results; PCA indicated tighter PCA clustering specially upon binding to PEP8 and PEP44, and RMSF data delineates some attenuation across the glycine-rich loop and ATP-binding residues, which highlighted the hypothesis of peptide binding might induce some compaction and constrained conformational freedom to the protein structure. These phenomena can cause some perturbations in the substrate access/binding, ending up with significant impairment in the GSK-3β catalytic functions. By contrast, PEP40 demonstrates increased local mobility and comparatively weaker complex stability.

Enthalpic contribution of protein-peptide biding was also highlighted through MMPBSA analysis, which results showed PEP44 and PEP8 form the strongest GSK-3β complexes (− 188.482 and − 184.404 kJ/mol), dominated by van der Waals forces, with notable electrostatic contributions. PEP36 binds strongly but suffers higher solvation penalties. PEP8 shows optimal SASA energy, while PEP40 forms the weakest complex, mainly stabilized by hydrophobic contacts. Together, our in silico pipeline identifies PEP8 and PEP44 as promising κ-casein-derived inhibitors that perturb GSK-3β’s active site architecture and dynamics. Ultimately, these peptides exemplify a rational route from food-derived sequences to multifunctional leads that may be tuned to counteract complex Alzheimer’s pathology.

## Supplementary Information

Below is the link to the electronic supplementary material.

Supplementary Material 1

## Data Availability

All the data generated or analyzed during this study are available in the manuscript and supporting information.
