# Supplementary material for: Rational design of k-casein peptides to modulate GSK-3B dynamics for Alzheimer’s therapy
Source: Sci Rep. 2026 Mar 9;16:12768. doi: 10.1038/s41598-026-42103-z (PMC13096195; doi:10.1038/s41598-026-42103-z)
Supplement: Supplementary file 1 — Supplementary Material 1 [file 41598_2026_42103_MOESM1_ESM.docx]

**Supporting Information**

**Rational Design of k-casein Peptides to Modulate GSK-3B Dynamics for Alzheimer’s Therapy**

Neda Moghaddam ^a^, Ali Ramazani ^*a,b,c^, Armin Zarei ^a^

*^a^The Organic Chemistry Research Laboratory (OCRL), Department of Chemistry, Faculty of Science, University of Zanjan, Zanjan, 45371-38791, Iran*

*^b^The Convergent Sciences & Technologies Laboratory (CSTL), Research Institute of Modern Biological Techniques (RIMBT), University of Zanjan, Zanjan, 45371-38791, Iran*

*^c^Universal Scientific Education and Research Network (USERN), University of Zanjan, Zanjan, 45371-38791, Iran*

*Corresponding author:

Ali Ramazani

Department of Chemistry, Faculty of Science, University of Zanjan, Zanjan, Iran.

Department of Biotechnology, Research Institute of Modern Biological Techniques (RIMBT), University of Zanjan, Zanjan, 45371-38791, Iran.

Email Addresses: [aliramazani@gmail.com](mailto:aliramazani@gmail.com) aliramazani@znu.ac.ir (A. Ramazani)

**Tabel S1.** The library of 48 mutant peptides and their safety profile.

| **Peptide NO.** | **Sequence** | **Allergenicity** | **Toxicity** |
| --- | --- | --- | --- |
| TEMPLATE | LALTLPFLGA |  |  |
| PEP1 | RARLLPELGA | NON-ALLERGEN | Non-Toxin |
| PEP2 | RAHLLPDLGA | NON-ALLERGEN | Non-Toxin |
| PEP3 | HARLLPHLGA | ALLERGEN |  |
| PEP4 | HAHLLPELGA | ALLERGEN |  |
| PEP5 | RARLLPDLGA | NON-ALLERGEN | Non-Toxin |
| PEP6 | RAHLLPHLGA | ALLERGEN |  |
| PEP7 | HARLLPELGA | ALLERGEN |  |
| PEP8 | HAHLLPDLGA | NON-ALLERGEN | Non-Toxin |
| PEP9 | RARLLPHLGA | NON-ALLERGEN | Non-Toxin |
| PEP10 | RAHLLPELGA | NON-ALLERGEN | Non-Toxin |
| PEP11 | HARLLPDLGA | ALLERGEN |  |
| PEP12 | HAHELPHLGA | NON-ALLERGEN | Non-Toxin |
| PEP13 | RARELPELGA | ALLERGEN |  |
| PEP14 | RAHELPDLGA | ALLERGEN |  |
| PEP15 | HARELPHLGA | NON-ALLERGEN | Non-Toxin |
| PEP16 | HAHELPELGA | ALLERGEN |  |
| PEP17 | RARELPDLGA | ALLERGEN |  |
| PEP18 | RAHELPHLGA | NON-ALLERGEN | Non-Toxin |
| PEP19 | HARELPELGA | ALLERGEN |  |
| PEP20 | HAHELPDLGA | ALLERGEN |  |
| PEP21 | RARELPHLGA | NON-ALLERGEN | Non-Toxin |
| PEP22 | RAHELPELGA | ALLERGEN |  |
| PEP23 | HARELPDLGA | ALLERGEN |  |
| PEP24 | HAHELPHLGA | NON-ALLERGEN | Non-Toxin |
| PEP25 | RARDLPELGA | ALLERGEN |  |
| PEP26 | RAHDLPDLGA | NON-ALLERGEN | Non-Toxin |
| PEP27 | HARDLPHLGA | NON-ALLERGEN | Non-Toxin |
| PEP28 | HAHDLPELGA | ALLERGEN |  |
| PEP29 | RARDLPDLGA | NON-ALLERGEN | Non-Toxin |
| PEP30 | RAHDLPHLGA | NON-ALLERGEN | Non-Toxin |
| PEP31 | HARDLPELGA | ALLERGEN |  |
| PEP32 | HAHDLPDLGA | ALLERGEN |  |
| PEP33 | RARDLPHLGA | NON-ALLERGEN | Non-Toxin |
| PEP34 | RARDLPELGA | ALLERGEN |  |
| PEP35 | HARDLPDLGA | ALLERGEN |  |
| PEP36 | HAHDLPHLGA | NON-ALLERGEN | Non-Toxin |
| PEP37 | RARILPELGA | NON-ALLERGEN | Non-Toxin |
| PEP38 | RAHILPDLGA | NON-ALLERGEN | Non-Toxin |
| PEP38 | HARILPHLGA | ALLERGEN |  |
| PEP40 | HAHILPELGA | NON-ALLERGEN | Non-Toxin |
| PEP41 | RARILPDLGA | ALLERGEN |  |
| PEP42 | RAHILPHLGA | ALLERGEN |  |
| PEP43 | HARILPELGA | ALLERGEN |  |
| PEP44 | HAHILPDLGA | NON-ALLERGEN | Non-Toxin |
| PEP45 | HARILPHLGA | ALLERGEN |  |
| PEP46 | RAHILPELGA | NON-ALLERGEN | Non-Toxin |
| PEP47 | HARILPDLGA | ALLERGEN |  |
| PEP48 | HAHILPHLGA | ALLERGEN |  |
